# A comprehensive nutritional survey of hospitalized patients: Results from nutritionDay 2016 in China

**DOI:** 10.1371/journal.pone.0194312

**Published:** 2018-03-22

**Authors:** Haifeng Sun, Li Zhang, Pianhong Zhang, Jianchun Yu, Weiming Kang, Shuli Guo, Wei Chen, Xuqi Li, Shufeng Wang, Lianzhen Chen, Jianxiong Wu, Zibin Tian, Xianghua Wu, Xiaosun Liu, Yinghua Liu, Xinying Wang

**Affiliations:** 1 Department of General Surgery, Jinling Hospital, Medical School of Nanjing University, Nanjing, China; 2 Department of clinical nutrition, School of Medicine, the Second Affiliated Hospital Zhejiang University, Hangzhou, China; 3 Peking Union Medical College Hospital, Beijing, China; 4 Department of General Surgery, The First Affiliated Hospital of Xi’an Jiaotong University, Xi’an, China; 5 National Cancer Center/Cancer hospital, Chinese Academy of Medical Sciences and Peking Union Medical College, Beijing, China; 6 Department of Gastroenterology, the Affiliated Hospital of Qingdao University, Qingdao, China; 7 The First Affiliated Hospital of Guangxi Medical University, Nanning, China; 8 Department of Gastrointestinal surgery, the First Affiliated Hospital Zhejiang University, Hangzhou, China; 9 Nutrition Department of People Liberation Army General Hospital of China, Beijing, China; National Yang-Ming University, TAIWAN

## Abstract

**Aims:**

Prevalence of malnutrition is a common and serious issue responsible for the morbidity and mortality rate among hospitalized patients. We aimed to provide an actual and comprehensive situation of the nutritional characteristics, nutritional support and the risk factors for malnutrition among hospitalized patients in China.

**Methods:**

We analyzed the data from nutritionDay audit 2016 in China. The international daylong cross-sectional survey was performed on November 10^th^, 2016 via filling out several questionnaires regarding information on patients’ illness, food intake history, weight change and nutritional care. Re-assessment of patients’ outcome questionnaire was performed 30 days later.

**Results:**

Total of 781 patients from 9 hospitals and 8 kinds of departments were enrolled in this report. Of these, malnutrition rate was 29.6%. Parenteral nutrition (251/344, 73.0%) was the primary nutrition support form in Chinese hospitals. However, 41.8% (136/325) of patients at nutritional risk or already diagnosed with malnutrition did not received any form of nutritional support, whereas 34.0% (155/456) well-nourished patients did. Patients with malnutrition had extended length of hospital stay and poor 30-day outcomes compared to well-nourished patients. Nutritional support could benefit nutritional risk or malnutrition patients, rather than well-nourished patients. Moreover, major lesion types, self-related health, food intake last week were independent risk factors of malnutrition (all *p*<0.05).

**Conclusions:**

Chinese hospital staff is generally lack of knowledge and awareness of malnutrition. Self-related health, major lesion types and food intake are associated with malnutrition.

## Introduction

Unbalanced nutritional status causes marginal extremes of unhealthy weight fluctuation. The excess of body weight, known as obesity, has become a current global problem that affects public health, and has received escalating public attention [1]. However, malnutrition, mostly recognized as a deficiency of energy, protein, or other nutrients, has always been neglected in clinical practice despite the fact that it also imposes a threat on public health. Indeed, malnutrition is not only a consequence of disease, but it can also exacerbate the progress of disease or even be causative [2]. In the past decades, a growing body of evidence demonstrated the negative effects of malnutrition, including immune dysfunction, rehabilitation delay, treatment failure, higher rate of infections and complications, and malnutrition may increase the duration of hospitalization, the readmission rate, and the mortality rate [2–5].

Despite the robust evidence of its life-threatening consequences, the prevalence of malnutrition is still a serious problem, especially in hospitalized patients. Previous studies showed that 10–50% of hospitalized patients were diagnosed with malnutrition, depending on the country, race, and diagnostic criteria [5–8]. The reason may be that hospitalized patients need more nutrients for their recovery than the general population [9], and that they may present with metabolic disorders or disease-induced reduction of food intake. Additionally, other factors can also contribute to malnutrition, such as age, pain, loss of taste or smell, main organ dysfunction, problems in chewing or swallowing, omission of essential nutrients, and psychological illness [8].

In China, clinical nutrition is a novel research field facing severe challenges. Comprehensive nutritional surveys in China are rare in last decade. Malnutrition in most hospitals is often missed in diagnosis due to unawareness or inaccurate nutritional screening and assessment. Moreover, inappropriate nutritional support, contrary to the suggested guidelines, is also common and this may further lead to deterioration of the patients’ nutritional status. Hence, there is an urgent need to perform a comprehensive study on malnutrition and nutritional support in China to help increase awareness about malnutrition and enhance the quality of nutritional care.

To begin to address this issue, nine hospitals from all over the country participated in the nutritionDay audit 2016 in China. The nutritionDay worldwide audit is a daylong cross-sectional survey performed annually in health care institutions. The prospective data mainly about patients’ illness and nutritional characteristics, food intake history, weight change, nutritional care and 30-day follow-up were collected and analyzed from heterogeneous medical settings: hospital wards, intensive care units (ICU) and nursing homes. The audit is hosted by the Medical University of Vienna, the European Society for Clinical Nutrition and Metabolism (ESPEN), and the Austrian Society for Clinical Nutrition. Nowadays, the nutritionDay has grown into a standard tool for determining the nutritional status and behavior of hospitalized patients [10].

In the present study, we aimed to provide data regarding the nutritional characteristics and the nutritional care of hospitalized patients in China, to try to identify potential risk factors associated with malnutrition, and to recognize the association between nutritional status and outcomes in hospitalized patients in China.

## Methods

### Study population

This observational survey was performed in patients from 9 Chinese hospitals (four located in east China, three located in north China, one located in south China and one located in northwest China) during the nutritionDay audit. The study was performed on November 10^th^, 2016. Hospitalized patients from all departments except ICU were enrolled in this survey. (1) Patients less than 7 years old, (2) patients admitted and discharged during the same calendar day, (3) patients who refused to answer the patient-specific questionnaire or refused to provide medical data, (4) and patients from the units whose participation rate is lower than 60% were excluded from the survey.

### Data collection procedure

The data were collected mainly using 3 coordinated questionnaires. (1) Patient-descriptions were completed by the unit staff in order to collect patients’ characteristics, disease-related information, and nutritional support information. (2) Patient questionnaires were completed by patients or caregivers (if necessary) in order to collect information on weight change, appetite, food intake history, mobility, and social contacts. (3) The outcome questionnaires were re-assessed by the unit staff on December 10^th^, 2016 in order to collect information on the 30-day outcomes, including discharge date, discharge diagnosis, current outcome status, and readmission. We followed up and calculated the total length of hospital stay (LOS) of 350 patients in Jinling Hospital and National Cancer Center/Cancer Hospital. All these questionnaires are available in https://www.nutritionday.org.

### Sample size and power calculation

The estimated sample size is n. The incidence of malnutrition in our previous study is approximately 29.3% [5]. According to the sample size estimate formula for the overall incidence of malnutrition in cross-sectional study (n = z_α/2_·*p*(1-*p*)/ δ^2^; α = 0.05, permissible error δ = 0.03), 451 patients is enough. None patients was lost during this cross-sectional study.

### Ethics

The nutritionDay audit was approved by the Ethical Committee of the Medical University of Vienna (EK407/2005) [11] and Chinese host hospital (Jinling hospital, code center number 1103) (2016NZKY-020-01). All the participants were volunteers and signed an informed consent before the audit. Hospitals and units were labeled with a numeric code, and participants were recorded with the initials of their name to protect patients’ privacy.

### Definitions

According to ESPEN consensus statement, individuals with a body mass index (BMI) <18.5 kg/m^2^ or unintentional weight loss (mandatory) >5% of habitual weight over 3 months are diagnosed with malnutrition [12]. Besides, patients diagnosed with malnutrition via nutritional screening or assessment tools were also included.

Major lesion types was confirmed mainly according to the primary discharge diagnosis or the primary admitting diagnosis for patients still in hospital.

The 30-day outcome status was assessed with the following options: death, still in hospital, transferred to another medical center, discharged home and rehabilitation. For further analysis, the overall 30-day outcome was dichotomized into 2 categories: favorable and poor outcome. The favorable outcome was defined as home discharge without re-admission, or rehabilitation. The rest were defined as poor outcome.

### Statistical analysis

All statistical analyses were performed using the Statistical Package for Social Sciences version 20.0 (SPSS Inc., Chicago, IL, USA). Data was reported as mean ± standard deviations (SD) for normally distributed variables. The Cox proportional hazards regression model was used for confirming the independent predictors for LOS. Odds ratios (OR) were described with 95% confidence intervals (CI). Multivariate linear regression models were used to identify the independent factors for malnutrition via a step-forward method. For all tests, *p*<0.05 was considered statistically significant.

## Results

### Basic demographic characteristics of hospitalized patients

The details of patients’ basic characteristics are shown in Table 1. A total of 781 hospitalized patients in 8 kinds of departments from 9 hospitals were enrolled in this report. Most of them were patients from the department of general surgery (45.7%), and gastroenterology and hepatology (internal medicine) (18.6%). The major lesion types [13] are mainly concentrated on cancer (n = 296, 37.9%), digestive organs (264, 33.8%) and neurological diseases (97, 12.4%). Nearly half of patients (45.5%) received operation after admission.

**Table 1:** Basic characteristics of hospitalized patients.

Characteristic	No. of Patients (Mean±SD)	No. of Patients (%)
Hospital	781	
Jinling Hospital		336 (43.0%)
Peking Union Medical College Hospital		111 (14.2%)
Second Affiliated Hospital of Zhejiang University		121 (15.5%)
People Liberation Army General Hospital of China		13 (1.7%)
First Affiliated Hospital of Xi’an Jiaotong University		81 (10.4%)
First Affiliated Hospital of Guangxi Medical University		30 (3.8%)
Affiliated Hospital of Qingdao University		34 (4.4%)
First Affiliated Hospital of Zhejiang University		23 (2.9%)
National Cancer Center/Cancer hospital		32 (4.1%)
Unit	781	
General surgery		357 (45.7%)
Cardiothoracic surgery		41 (5.2%)
Neurosurgery		42 (5.4%)
Gastroenterology and hepatology		145 (18.6%)
Oncology		76 (9.7%)
Neurology		63 (8.1%)
Nephrology		44 (5.6%)
Geriatrics		13 (1.7%)
Sex	780	
Male		469 (60.1%)
Female		311 (39.9%)
Age, years	781 (55.4±16.2)	
≤65 years		559 (71.6%)
>65 years		222 (28.4%)
Weight, kg	781 (63.2±12.5)	
Height, cm	781 (165.9±8.3)	
BMI, kg/m2	781 (22.9±3.7)	
<18.5		95 (12.2%)
18.5–25		472 (60.4%)
>25		214 (27.4%)
Major lesion types	781	
Cancer		296 (37.9%)
Neurological disease		97 (12.4%)
Digestive disease		264 (33.8%)
Endocrine/nutritional/metabolic disease		13 (1.7%)
Cardiovascular disease		16 (2.0%)
Respiratory disease		15 (1.9%)
Genitourinary disease		40 (5.1%)
Others		40 (5.1%)
Operation after admission	739	
Yes, elective operation		324 (43.8%)
Yes, emergency operation		12 (1.6%)
No		403 (54.5%)
Ever stayed in ICU	756	
Yes		127 (16.8%)
No		629 (83.2%)
End-stage disease	781	
Yes		27 (3.5%)
No		650 (83.2%)
I don’t know		104 (13.3%)
Motility	781	
Walk without assistance		609 (78.0%)
Walk with assistance		108 (13.8%)
Bedridden		64 (8.2%)
Self-rated health	781	
Very good		52 (6.7%)
Good		299 (38.3%)
Fair		342 (43.8%)
Poor		77 (9.9%)
Very poor		11 (1.4%)
No. of drugs before admission/day	781	
0		277 (35.5%)
1–2		254 (32.5%)
3–5		179 (22.9%)
>5		42 (5.4%)
I don’t know		29 (3.7%)
Medical insurance	753	
Yes, private insurance only		33 (4.4%)
Yes, public insurance only		546 (72.5%)
Yes, both		42 (5.6%)
No		104 (13.8%)
I prefer not to answer		28 (3.7%)

The mean age was 55.4 years old, and there into, 222 (28.4%) patients were elderly [14]. The mean BMI was 22.9 kg/m^2^. According to the Global Health Organization guidelines for the BMI classification, 95 patients (12.2%) were underweight (<18.5 kg/m^2^). The majority of patients (78.0%) could walk without assistance. About 9.9% of the patients reported poor feeling and 1.4% of the patients reported very poor feeling [15]. Almost 90% of hospitalized patients had medical insurance in China.

### Nutrition related characteristics

**Fig 1:**
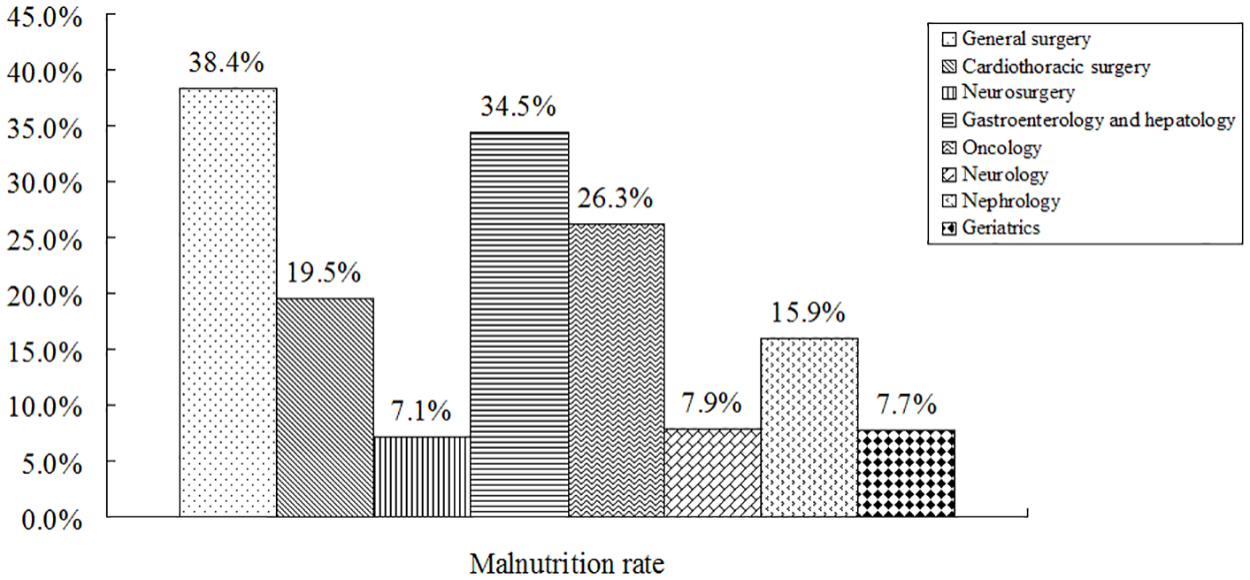
Prevalence of malnutrition by departments in Chinese hospitals.

Of all participants, the malnutrition rate was 29.6%, and higher in the department of general surgery (38.4%) and the department of gastroenterology and hepatology (34.5%). The prevalence of malnutrition by departments was detailed in Fig 1.

344 patients (44%) received nutritional support, and among these, parenteral nutrition (PN) was the most commonly used method. 334 patients (42.8%) experienced weight loss within the last 3 months, and about 2/3 of these patients lost weight unintentionally. 774 patients provided information on their food intake last week. About 24.8% of the patients ate less than normal last week. On nutritionDay, about 68.7% of the patients did not eat full lunch or dinner and 38.7% patients ate nothing. The median LOS among the 350 hospitalized patients was 13 days (not showed in Table 2), ranging from 1 to 157 days. Patients’ nutritional characteristics are detailed in Table 2.

**Table 2:** Nutritional characteristics of hospitalized patients.

Characteristic	No. of Patients (range)	No. of Patients (%)
Malnutrition	781	
Yes		231 (29.6%)
No		550 (70.4%)
Nutritional support	781	
Yes		344 (44.0%)
No		437 (56.0%)
Nutritional support form	781	
ONS		34 (4.4%)
EN		40 (5.1%)
PN		144 (18.4%)
Multi-forms		126 (16.1%)
None		437 (56.0%)
Weight loss within last 3 months	781	
Yes, unintentionally		222 (28.4%)
Yes, intentionally		112 (14.3%)
Stable weight		343 (43.9%)
No, weight gain		46 (5.9%)
I don’t know		58 (7.4%)
Food intake last week	774	
More than normal		17 (2.2%)
Normal		565 (73.0%)
About 3/4 of normal		57 (7.4%)
About half of normal		67 (8.7%)
About a quarter to nearly nothing		68 (8.8%)
Proportion of lunch/dinner eaten on nutritionDay	780	
About all		244 (31.3%)
1/2		144 (18.5%)
1/4		90 (11.5%)
Nothing		302 (38.7%)
LOS, days	350 (1–157)	

ONS, oral nutritional supplementation; EN, enteral nutrition; PN, parenteral nutrition; LOS, length of stay

### Inappropriate nutritional therapy in Chinese hospitals

**Fig 2:**
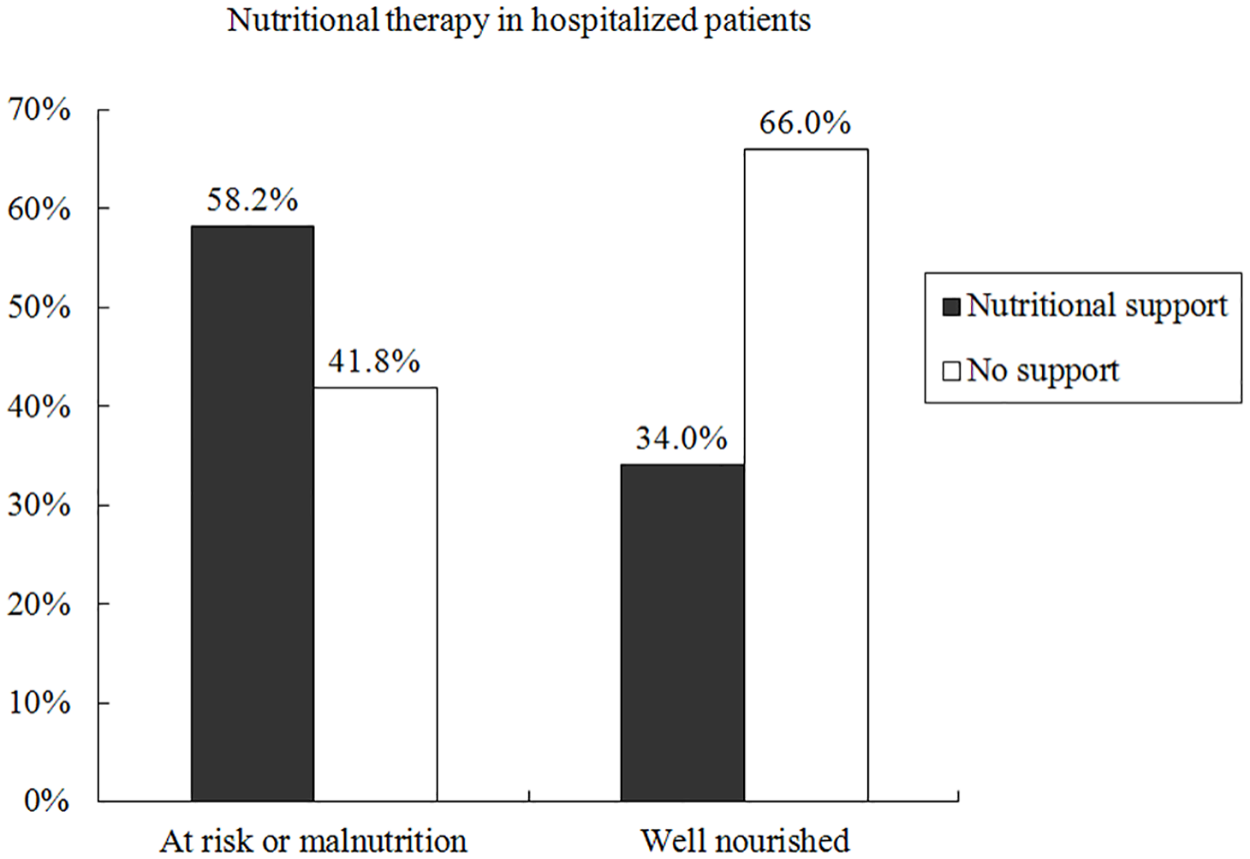
Nutritional therapy in Chinese hospitals.

Of all 781 hospitalized patients, 231 patients (29.6%) were diagnosed with malnutrition, 94 patients (12.0%) were at nutrition risk according to nutrition risk screening (NRS 2002) [16], and 456 patients (58.4%) were well nourished. According to guidelines patients at nutritional risk or diagnosed with malnutrition should receive a nutritional care plan and these patients may benefit from the nutritional therapy [16]. However, among 325 patients at nutritional risk or already diagnosed with malnutrition, 136 patients (41.8%) did not receive any form of nutritional support. Among 456 well-nourished patients, 155 patients (34.0%) received improper nutritional support. These details are shown in Fig 2.

### Association between malnutrition and poor 30-day outcome

**Fig 3:**
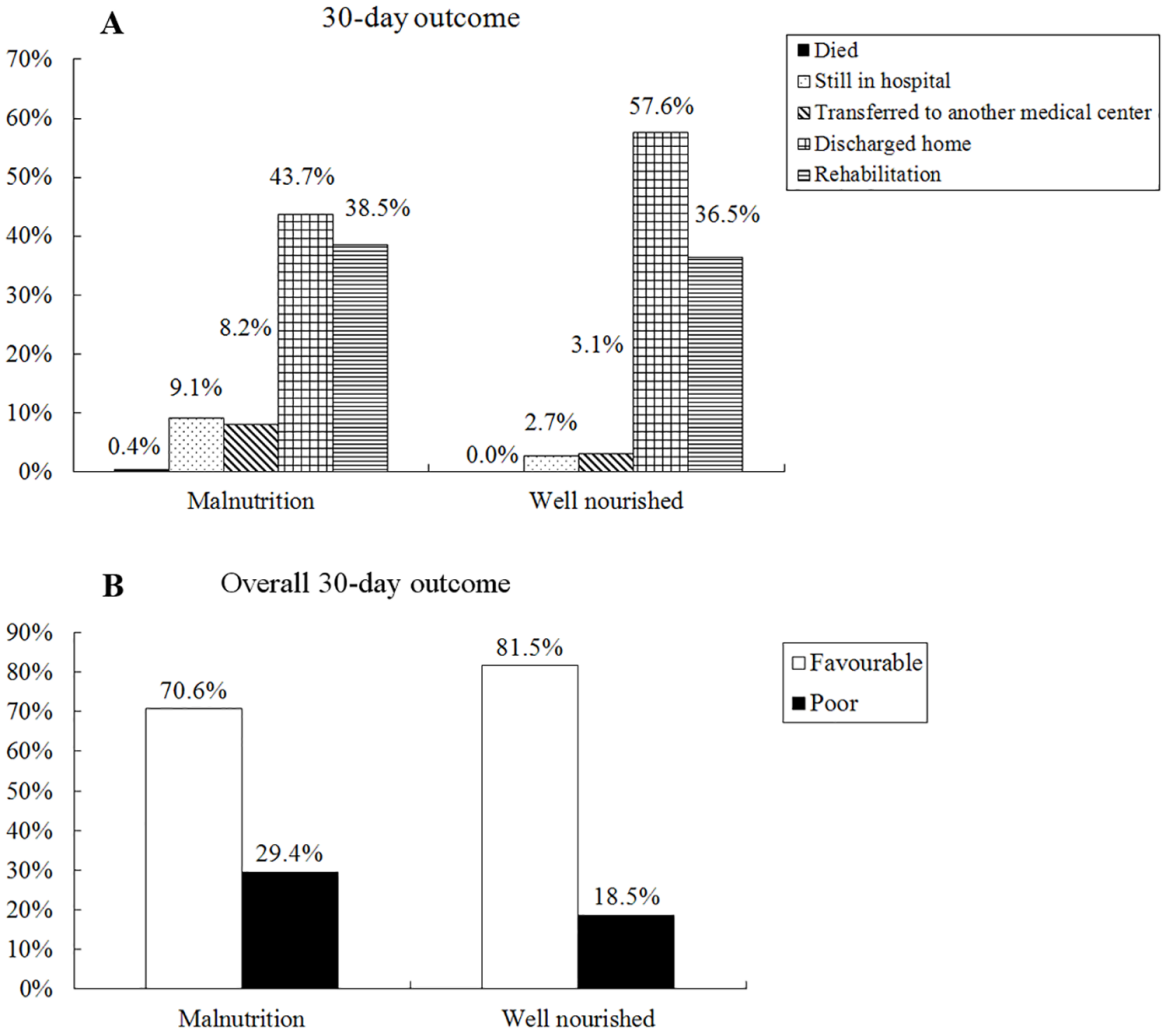
Relationship between nutritional status and outcomes in Chinese hospitalized patients. Overall 30-day outcomes: favorable, defined as rehabilitation or home discharge without re-admission; poor, defined as died, still in hospital, transferred to another medical center or home discharge with re-admission within 30 days.

The relationship between malnutrition and the 30-day outcome was shown in Fig 3. Compared to well-nourished patients, patients with malnutrition presented similar mortality (0.4% vs. 0.0%, *p* = 0.123), longer hospital stay rate (9.1% vs. 2.7%, *p*<0.001) and higher transfer rate (8.2% vs. 3.1%, *p* = 0.002), lower discharge rate (43.7% vs. 57.6%, *p*<0.001) and similar rehabilitation rate (38.5% vs. 36.5%, *p* = 0.601). According to overall 30-day outcome, patients with malnutrition were more likely to achieve poor outcome compared to well-nourished patients (29.4% vs. 18.5%, *p*<0.001).

### Association between nutritional support and 30-day outcome

**Fig 4:**
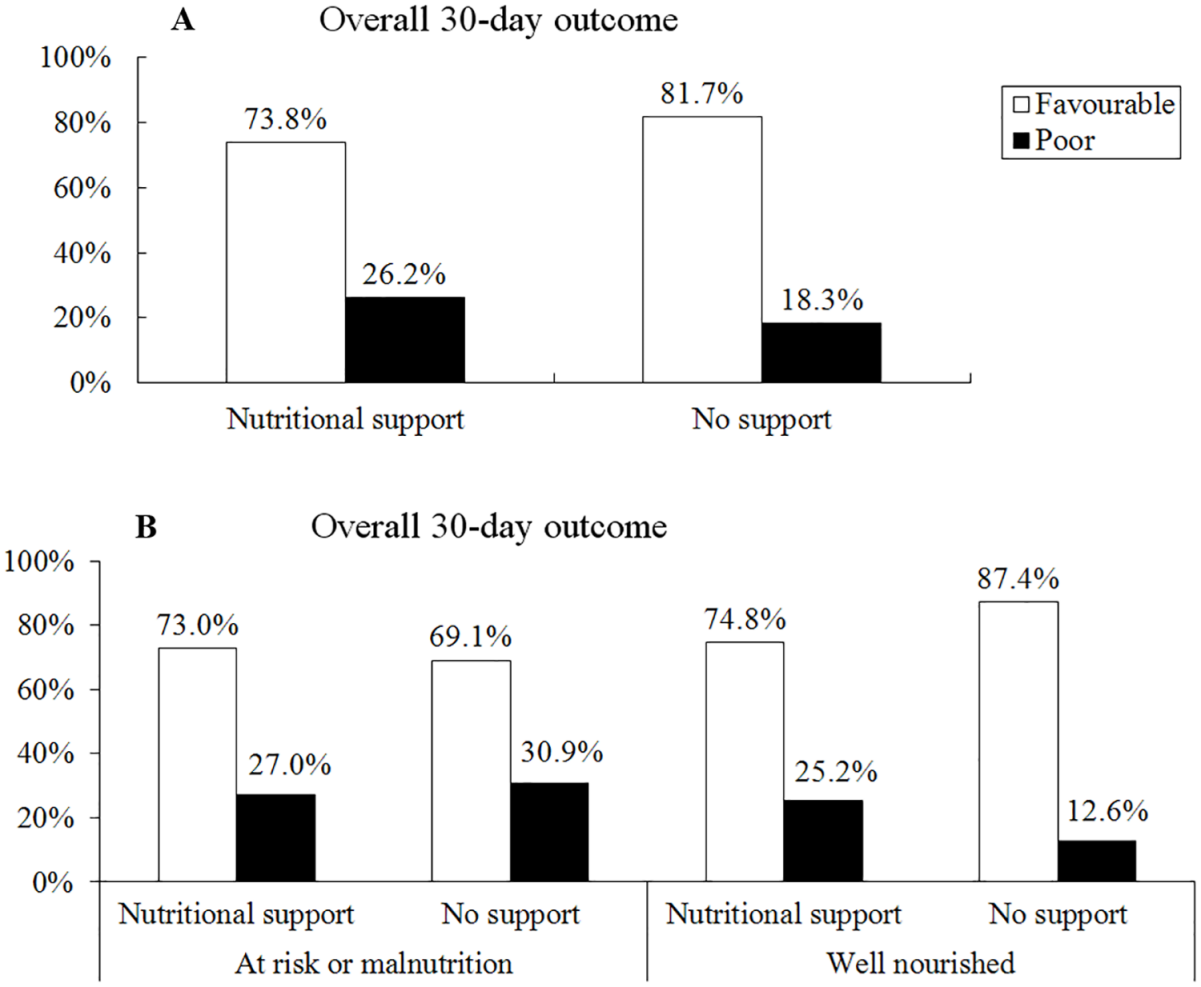
Relationship between nutritional support and overall 30-day outcomes in Chinese hospitalized patients. Overall 30-day outcomes: favorable, defined as rehabilitation or home discharge without re-admission; poor, defined as died, still in hospital, transferred to another medical center or home discharge with re-admission within 30 days.

The relationship between nutritional support and 30-day outcome was shown in Fig 4. Patients with nutritional support were more likely to achieve poor outcome compared to patients without nutritional support (26.2% vs. 18.3%, *p* = 0.008). Nutritional support slightly improved the overall 30-day outcomes for patients with nutrition risk or malnutrition (73.0% vs. 69.1%, *p* = 0.443), whereas associated with poor overall 30-day outcomes in well-nourished patients (25.2% vs. 12.6%, *p*<0.001).

### Risk factors associated with the length of hospital stay

Results are presented in detail in Table 3. Patients with malnutrition had longer LOS compared to well-nourished patients (*p* = 0.019). Besides, significant difference of LOS was also found among patients with different BMI classifications (*p* = 0.038), different major lesion types (*p* = 0.032), ever stayed in ICU or not (*p*<0.001), different motility groups (*p* = 0.021), nutritional support or not (*p*<0.001) and depending on the proportion of lunch/dinner eaten on nutritionDay (*p* = 0.016). Then, all variables above were enrolled in a step-forward logistic regression approach of Cox proportional hazards regression models. Our results showed ever stayed in ICU (*p*<0.001) and nutritional support (*p*<0.001) were independent factors of prolonged LOS of hospitalized patients.

**Table 3:** Risk factors for LOS in Jinling hospital and National Cancer Center/Cancer hospital.

Characteristic	LOS, days	Univariate analysis		Multivariate analysis	
	Median (range)	OR (95% CI)	*p*	OR (95% CI)	*p*
Sex			0.534		
Male	12 (1–157)	1			
Female	14 (1–78)	1.071 (0.863–1.329)			
Age			0.244		
≤65 years	13 (1–157)	1			
>65 years	12 (2–56)	1.157 (0.905–1.478)			
BMI, kg/m2			**0.038**		*
<18.5	19 (1–63)	1			
18.5–25	12 (1–157)	1.496 (1.049–2.135)			
>25	11 (2–99)	1.637 (1.120–2.392)			
Major lesion types			**0.032**		*
Cancer	14 (2–78)	1			
Neurological disease	11 (1–94)	1.295 (0.981–1.711)			
Digestive disease	15 (1–78)	0.914 (0.677–1.235)			
Endocrine/nutritional/metabolic disease	8 (7–17)	1.811 (0.574–5.715)			
Cardiovascular disease	18 (5–33)	0.900 (0.516–1.570)			
Respiratory disease	11 (2–99)	0.837 (0.455–1.540)			
Genitourinary disease	9 (6–35)	1.468 (1.018–2.117)			
Others	14.5 (8–157)	0.469 (0.216–1.018)			
Ever stayed in ICU			**<0.001**		**<0.001**
Yes	15 (5–157)	1		1	
No	11 (1–78)	1.809 (1.397–2.344)		1.805 (1.430–2.293)	
End-stage disease			0.992		
Yes	13.5 (1–94)	1			
No	13 (1–157)	1.023 (0.615–1.700)			
I don’t know	12 (2–78)	1.009 (0.589–1.729)			
Motility			**0.021**		*
Walk without assistance	11 (1–78)	1.655 (1.102–2.487)			
Walk with assistance	14 (4–99)	1.289 (0.808–2.056)			
Bedridden	15 (1–157)	1			
Self-rated health			0.442		
Very good	12 (4–43)	1.493 (0.531–4.194)			
Good	13 (1–53)	1.350 (0.553–3.297)			
Fair	12 (1–157)	1.150 (0.470–2.813)			
Poor	12 (2–33)	1.530 (0.600–3.902)			
Very poor	18 (14–22)	1			
Malnutrition			**0.019**		*
Yes	16 (1–63)	1			
No	11 (1–157)	1.362 (1.052–1.764)			
Nutritional support			**<0.001**		**<0.001**
Yes	16 (1–157)	1		1	
No	11 (1–94)	1.727 (1.375–2.169)		1.808 (1.426–2.291)	
Weight loss within last 3 months			0.426		
Yes, unintentionally	12 (1–63)	1			
Yes, intentionally	16 (4–78)	0.764 (0.532–1098)			
Stable weight	12 (1–157)	1.033 (0.789–1.353)			
No, weight gain	14 (2–44)	0.882 (0.544–1.428)			
I don’t know	12 (4–43)	1.069 (0.723–1.581)			
Food intake last week			0.314		
More than normal	9 (7–31)	1.626 (0.693–3.812)			
Normal	12 (1–157)	1.330 (0.859–2.060)			
About 3/4 of normal	14 (2–78)	1.025 (0.587–1.789)			
About half of normal	15 (3–56)	1.042 (0.607–1.788)			
About a quarter to nearly nothing	18.5 (3–38)	1			
Proportion of lunch/dinner eaten on nutritionDay			**0.016**		*
About all	10 (1–94)	1.548 (1.183–2.026)			
1/2	11 (2–78)	1.319 (0.976–1.783)			
1/4	14 (5–42)	1.214 (0.842–1.751)			
Nothing	14 (1–157)	1			

*Forward LR: not in the final step

### Relationship between malnutrition and patients’ characteristics

The univariate analysis showed that the rate of malnutrition was significantly different between sexes (*p* = 0.022), major lesion types (*p*<0.001), motility (*p* = 0.030), self-rated health (*p*<0.001), and food intake last week (*p*<0.001). There was no statistical relationship between the malnutrition rate and age, ICU stay, and disease stage groups. According to multivariate analysis, the major lesion types (*p*<0.001), self-rated health (*p* = 0.003), and food intake last week (*p*<0.001) were independent risk factors of malnutrition in hospitalized patients in China. The results are presented in detail in Table 4.

**Table 4:** Relationship between malnutrition and patients’ characteristics.

Characteristic	Univariate analysis			Multivariate analysis	
	Malnutrition	Well nourtished	*p*	OR (95% CI)	*p*
Sex			**0.022**		*
Male	124 (26.4%)	345 (73.6%)			
Female	106 (34.1%)	205 (65.9%)			
Age			0.451		*
≤65 years	161 (28.8%)	398 (71.2%)			
>65 years	70 (31.5%)	152 (68.5%)			
Major lesion types			**<0.001**		**<0.001**
Cancer	102 (34.5%)	194 (65.5%)		1	
Neurological disease	7 (7.2%)	90 (92.8%)		0.145 (0.062–0.338)	
Digestive disease	100 (37.9%)	164 (62.1%)		1.069 (0.736–1.553)	
Endocrine/nutritional/metabolic disease	2 (15.4%)	11 (84.6%)		0.385 (0.080–1.846)	
Cardiovascular disease	1 (6.2%)	15 (93.8%)		0.121 (0.015–0.962)	
Respiratory disease	2 (13.3%)	13 (86.7%)		0.466 (0.094–2.309)	
Genitourinary disease	6 (15.0%)	34 (85.0%)		0.375 (0.136–1.040)	
Others	11 (27.5%)	29 (72.5%)		0.832 (0.382–1.814)	
Ever stayed in ICU			0.826		*
Yes	187 (29.7%)	442 (70.3%)			
No	39 (30.7%)	88 (69.3%)			
End-stage disease			0.684		*
Yes	10 (37.0%)	17 (63.0%)			
No	191 (29.4%)	459 (70.6%)			
I don’t know	30 (28.8%)	74 (71.2%)			
Motility			**0.03**		*
Walk without assistance	170 (27.9%)	439 (72.1%)			
Walk with assistance	33 (30.6%)	75 (69.4%)			
Bedridden	28 (43.8%)	36 (56.2%)			
Self-rated health			**<0.001**		**0.003**
Very good	9 (17.3%)	43 (82.7%)		1	
Good	69 (23.1%)	230 (76.9%)		2.129 (0.894–5.067)	
Fair	114 (33.3%)	228 (66.7%)		2.966 (1.256–7.004)	
Poor	32 (41.6%)	45 (58.4%)		4.500 (1.701–11.906)	
Very poor	7 (63.6%)	4 (36.4%)		12.353 (2.424–62.956)	
Food intake last week			**<0.001**		**<0.001**
More than normal	4 (23.5%)	13 (76.5%)		1	
Normal	129 (22.8%)	436 (77.2%)		0.658 (0.200–2.163)	
About 3/4 of normal	21 (36.8%)	36 (63.2%)		1.060 (0.284–3.959)	
About half of normal	32 (47.8%)	35 (52.2%)		1.720 (0.475–6.230)	
About a quarter to nearly nothing	41 (60.3%)	27 (39.7%)		2.077 (0.569–7.587)	

Univariate analysis: chi-square test; Multivariate analysis: binary logistic regression analysis

*Forward LR: not in the final step

## Discussion

The present study is the first nutritional survey among hospitalized patients during the past ten years that covers north, east, south, and northwest areas of China that provides a collection of current data reflecting the situation of clinical nutrition in China. The results showed that malnutrition is still a common and severe problem affecting patients’ outcome in Chinese hospitals. There were numerous nonstandard nutritional therapies in contrast to implemented guidelines. Moreover, major lesion types, self-rated health, food intake last week were risk factors of malnutrition.

The nutritionDay audit is the largest and most authoritative ongoing nutritional survey among hospitalized patients in the world aiming to fight malnutrition and to ameliorate nonstandard nutritional care in health care institutions. Cumulative findings reported consistently that nutritional characteristics were related to hospitalized patients’ health and outcomes but they have been always neglected during clinical practice [2,8,17]. Therefore, malnutrition is still very common in hospital settings, and it has been well documented in previous studies that 10%-50% hospitalized patients suffer from malnutrition [6–8,18]. One probable reason of this variation may be geographic distribution of the population that malnutrition rate varies greatly in different countries despite sharing the same criteria [5,18–20].

In the present report, the malnutrition rate in Chinese hospitals was 29.6%, similar to previous reports from 3 hospitals (29.3%) in China [5], Germany (27.4%) [18] and other European countries (27%) [2]. However, the malnutrition rate between the 9 Chinese hospitals studied here varied considerably. This was, at least in part, due to 2 reasons: first, different diagnostic accuracy (nutritional screening tools, awareness of malnutrition), and second, uneven efficacy of nutritional care. Moreover, the malnutrition rate varied significantly among different departments. It was not surprising that the malnutrition rate was higher in the department of general surgery and the department of gastroenterology and hepatology because surgery and the dysfunction of digestive system may result in metabolic disorders and dietary changes [8]. However, the low malnutrition rate observed in geriatrics is surprising [21]. The small sample size (only 13 patients) may explain this result; nonetheless, we hypothesized that elderly patients were more likely to suffer from malnutrition because of poor physical and dietary conditions. Accordingly, our results showed that patients older than 65 years old had higher malnutrition rate.

According to guidelines, patients at nutritional risk or already diagnosed with malnutrition should receive a nutrition care plan and these patients can benefit from nutritional therapy [16,22]. Consistently, nutritional therapy in the present study was compared between two classifications of hospitalized patients: at risk of malnutrition or diagnosed with malnutrition and well-nourished, rather than malnutrition and non-malnutrition as in previous studies [5,23]. Obviously, over 40% of patients at nutritional risk or already coded with malnutrition in need of nutritional intervention were neglected, even in those who could not eat enough meal. Then, about one third of well-nourished patients who had no nutritional requirements unexpectedly received at least one form of nutritional support in Chinese hospitals. Additionally, PN remains the overwhelming nutritional support form, though guidelines recommend early enteral nutrition (EN) in most cases [22]. These illegal phenomena demonstrate the lack of knowledge and awareness on malnutrition among Chinese hospital staff, and therefore nonstandard nutritional support against guidelines is prevalent in Chinese hospitals.

The close relationship between poor outcomes and malnutrition has been well established in many conditions, such as increased mortality [24], prolonged LOS [5,25], and increased readmission [4,25]. Consistent with these results, patients with malnutrition showed higher rate of mortality, longer hospital stay and higher transfer rate, also showed lower discharge rate compared to well-nourished patients in China hospitals. It was not surprising that patients who ate less on nutritionDay had longer LOS in our study as well. Consistent with that, food intake reduction is always associated with longer LOS and higher mortality [26]. It is noteworthy that patients with nutritional support also had significantly longer LOS than those without support, in accordance with a previous study [5]. However, we believe that this relationship is an artifact due to the bias stemming from the close relationship between malnutrition and nutritional support. Moreover, malnutrition is undeniably associated with increased cost due to longer hospital stay and more intensive treatment [27]. Fortunately, the coverage of medical insurance reaches up to nearly 90% in China.

Identification of predisposing factors for malnutrition may contribute to identify patients at nutritional risk [2,28]. Traditionally, malnutrition is mostly caused by insufficient energy, protein, or other essential nutrients intake for a prolonged period of time. However, evidence showed that hospitalized patients often received less nutritional care than expected because of lack of training and awareness of the hospital staff [29]. Consistently, the reduction of food intake last week consisted independent factors for malnutrition among Chinese hospitalized patients. Besides, a substantial number of studies indicates that malnutrition is more frequent in patients with cancer [30], digestive disease [31], and nervous system disease [32]. Our results showed that mainly patients with cancer and digestive disease, but not those with neurological disease, had high risk for malnutrition. The latter one is, at least in part, due to small sample size. Moreover, self-rated health was another risk factor for malnutrition in this work, similar to a previous study [15]. The report of this factor is advantageous because it is a simple and efficient way to reflect the physical and psychological well-being of patients. However, it is subjective and therefore prone to bias. However, inconsistent with previous knowledge, there was no significant relationship between malnutrition and age groups in our study. We speculate that the probable explanation may be that: firstly, the age-distribution of patients was not even enough in our study; secondly, elderly patients with malnutrition tended not to actively participate in the investigation because of their weakness and low literacy ability.

We report some limitations of this study. First, the innate limitation of the day-cross snapshot survey is prone to omit some potential long-term confounders. Second, the sample size may not enough to be representative of the entire Chinese hospitalized population. Third, hospitals and units sample size varies extremely and this may lead to a statistical bias. Four, the heterogeneity of disease and variable severity of disease in the same unit are neglected. Last, hospitals and departments with high malnutrition rate or interested in clinical nutrition are more likely to participate in this study, which may lead to a data bias.

## Conclusions

In summary, the prevalence of malnutrition is about 30% and associated with poor 30-day outcomes in Chinese hospitals. Inappropriate nutritional therapy is a common and urgent problem that needs to be solved. Specific nutritional interventions should be considered for patients with poor self-rated health, critical lesion types, and recent food intake reduction. Moreover, further studies should focus on the risk factors of food intake loss and on identifying efficient methods for protecting patients from malnutrition.

## Supporting information

S1 Table Patient-characteristic questionnaire. (PDF)

S2 Table Patient-description questionnaire. (PDF)

S3 Table Patient 30-day outcomes questionnaire. (PDF)

## Abbreviations

BMI: body mass index
CI: confidence intervals
EN: enteral nutrition
ESPEN: European Society for Clinical Nutrition and Metabolism
ICU: intensive care units
LOS: length of hospital stay
OR: odds ratios
PN: parenteral nutrition
SD: standard deviation

## References

[1] Lee SJ, Shin SW (2017) Mechanisms, Pathophysiology, and Management of Obesity. N Engl J Med 376: 1491–1492. 10.1056/NEJMc1701944 28406283

[2] Schindler K, Pernicka E, Laviano A, Howard P, Schutz T, et al (2010) How nutritional risk is assessed and managed in European hospitals: a survey of 21,007 patients findings from the 2007–2008 cross-sectional nutritionDay survey. Clin Nutr 29: 552–559. doi: 10.1016/j.clnu.2010.04.001 20434820 10.1016/j.clnu.2010.04.001

[3] Marco J, Barba R, Zapatero A, Matia P, Plaza S, et al (2011) Prevalence of the notification of malnutrition in the departments of internal medicine and its prognostic implications. Clin Nutr 30: 450–454. doi: 10.1016/j.clnu.2010.12.005 21300420 10.1016/j.clnu.2010.12.005

[4] Agarwal E, Ferguson M, Banks M, Batterham M, Bauer J, et al (2013) Malnutrition and poor food intake are associated with prolonged hospital stay, frequent readmissions, and greater in-hospital mortality: results from the Nutrition Care Day Survey 2010. Clin Nutr 32: 737–745. doi: 10.1016/j.clnu.2012.11.021 23260602 10.1016/j.clnu.2012.11.021

[5] Zheng H, Huang Y, Shi Y, Chen W, Yu J, et al (2016) Nutrition Status, Nutrition Support Therapy, and Food Intake are Related to Prolonged Hospital Stays in China: Results from the NutritionDay 2015 Survey. Ann Nutr Metab 69: 215–225. doi: 10.1159/000451063 27894108 10.1159/000451063

[6] McWhirter JP, Pennington CR (1994) Incidence and recognition of malnutrition in hospital. Bmj 308: 945–948. 8173401 10.1136/bmj.308.6934.945 PMC2539799

[7] Mulu H, Hamza L, Alemseged F (2016) Prevalence of Malnutrition and Associated Factors among Hospitalized Patients with Acquired Immunodeficiency Syndrome in Jimma University Specialized Hospital, Ethiopia. Ethiop J Health Sci 26: 217–226. 27358542 10.4314/ejhs.v26i3.4 PMC4913189

[8] Norman K, Pichard C, Lochs H, Pirlich M (2008) Prognostic impact of disease-related malnutrition. Clin Nutr 27: 5–15. doi: 10.1016/j.clnu.2007.10.007 18061312 10.1016/j.clnu.2007.10.007

[9] Navarro DA, Boaz M, Krause I, Elis A, Chernov K, et al (2016) Improved meal presentation increases food intake and decreases readmission rate in hospitalized patients. Clin Nutr 35: 1153–1158. doi: 10.1016/j.clnu.2015.09.012 26627844 10.1016/j.clnu.2015.09.012

[10] Schindler K, Pichard C, Sulz I, Volkert D, Streicher M, et al (2016) nutritionDay: 10 years of growth. Clin Nutr. 10.1016/j.clnu.2016.11.004 27916337

[11] Schindler K, Themessl-Huber M, Hiesmayr M, Kosak S, Lainscak M, et al (2016) To eat or not to eat? Indicators for reduced food intake in 91,245 patients hospitalized on nutritionDays 2006–2014 in 56 countries worldwide: a descriptive analysis. Am J Clin Nutr 104: 1393–1402. doi: 10.3945/ajcn.116.137125 27733401 10.3945/ajcn.116.137125

[12] Cederholm T, Bosaeus I, Barazzoni R, Bauer J, Van Gossum A, et al (2015) Diagnostic criteria for malnutrition—An ESPEN Consensus Statement. Clin Nutr 34: 335–340. doi: 10.1016/j.clnu.2015.03.001 25799486 10.1016/j.clnu.2015.03.001

[13] Hiesmayr M, Frantal S, Schindler K, Themessl-Huber M, Mouhieddine M, et al (2015) The Patient- And Nutrition-Derived Outcome Risk Assessment Score (PANDORA): Development of a Simple Predictive Risk Score for 30-Day In-Hospital Mortality Based on Demographics, Clinical Observation, and Nutrition. PLoS One 10: e0127316 doi: 10.1371/journal.pone.0127316 26000634 10.1371/journal.pone.0127316 PMC4441510

[14] Stott DJ, Rodondi N, Kearney PM, Ford I, Westendorp RG, et al (2017) Thyroid Hormone Therapy for Older Adults with Subclinical Hypothyroidism. N Engl J Med. 10.1056/NEJMc1709989 28976862

[15] Lainscak M, Farkas J, Frantal S, Singer P, Bauer P, et al (2014) Self-rated health, nutritional intake and mortality in adult hospitalized patients. Eur J Clin Invest 44: 813–824. doi: 10.1111/eci.12300 25039263 10.1111/eci.12300

[16] Kondrup J, Allison SP, Elia M, Vellas B, Plauth M (2003) ESPEN guidelines for nutrition screening 2002. Clin Nutr 22: 415–421. 12880610 10.1016/s0261-5614(03)00098-0

[17] Singh H, Watt K, Veitch R, Cantor M, Duerksen DR (2006) Malnutrition is prevalent in hospitalized medical patients: are housestaff identifying the malnourished patient? Nutrition 22: 350–354. doi: 10.1016/j.nut.2005.08.009 16457988 10.1016/j.nut.2005.08.009

[18] Pirlich M, Schutz T, Norman K, Gastell S, Lubke HJ, et al (2006) The German hospital malnutrition study. Clin Nutr 25: 563–572. doi: 10.1016/j.clnu.2006.03.005 16698132 10.1016/j.clnu.2006.03.005

[19] Correia MI, Campos AC (2003) Prevalence of hospital malnutrition in Latin America: the multicenter ELAN study. Nutrition 19: 823–825. 14559314 10.1016/s0899-9007(03)00168-0

[20] Edington J, Boorman J, Durrant ER, Perkins A, Giffin CV, et al (2000) Prevalence of malnutrition on admission to four hospitals in England. The Malnutrition Prevalence Group. Clin Nutr 19: 191–195. doi: 10.1054/clnu.1999.0121 10895110 10.1054/clnu.1999.0121

[21] Cereda E, Klersy C, Hiesmayr M, Schindler K, Singer P, et al (2017) Body mass index, age and in-hospital mortality: The NutritionDay multinational survey. Clin Nutr 36: 839–847. doi: 10.1016/j.clnu.2016.05.001 27236599 10.1016/j.clnu.2016.05.001

[22] McClave SA, Taylor BE, Martindale RG, Warren MM, Johnson DR, et al (2016) Guidelines for the Provision and Assessment of Nutrition Support Therapy in the Adult Critically Ill Patient: Society of Critical Care Medicine (SCCM) and American Society for Parenteral and Enteral Nutrition (A.S.P.E.N.). JPEN J Parenter Enteral Nutr 40: 159–211. doi: 10.1177/0148607115621863 26773077 10.1177/0148607115621863

[23] Zhang L, Wang X, Huang Y, Gao Y, Peng N, et al (2013) NutritionDay 2010 audit in Jinling hospital of China. Asia Pac J Clin Nutr 22: 206–213. doi: 10.6133/apjcn.2013.22.2.18 23635363 10.6133/apjcn.2013.22.2.18

[24] Asiimwe SB, Muzoora C, Wilson LA, Moore CC (2015) Bedside measures of malnutrition and association with mortality in hospitalized adults. Clin Nutr 34: 252–256. doi: 10.1016/j.clnu.2014.03.013 24755235 10.1016/j.clnu.2014.03.013

[25] Lim SL, Ong KC, Chan YH, Loke WC, Ferguson M, et al (2012) Malnutrition and its impact on cost of hospitalization, length of stay, readmission and 3-year mortality. Clin Nutr 31: 345–350. doi: 10.1016/j.clnu.2011.11.001 22122869 10.1016/j.clnu.2011.11.001

[26] Hiesmayr M, Schindler K, Pernicka E, Schuh C, Schoeniger-Hekele A, et al (2009) Decreased food intake is a risk factor for mortality in hospitalised patients: the NutritionDay survey 2006. Clin Nutr 28: 484–491. doi: 10.1016/j.clnu.2009.05.013 19573957 10.1016/j.clnu.2009.05.013

[27] Mitchell H, Porter J (2016) The cost-effectiveness of identifying and treating malnutrition in hospitals: a systematic review. J Hum Nutr Diet 29: 156–164. doi: 10.1111/jhn.12308 25808187 10.1111/jhn.12308

[28] Heyland DK, Dhaliwal R, Jiang X, Day AG (2011) Identifying critically ill patients who benefit the most from nutrition therapy: the development and initial validation of a novel risk assessment tool. Crit Care 15: R268 doi: 10.1186/cc10546 22085763 10.1186/cc10546 PMC3388687

[29] Kondrup J, Johansen N, Plum LM, Bak L, Larsen IH, et al (2002) Incidence of nutritional risk and causes of inadequate nutritional care in hospitals. Clin Nutr 21: 461–468. 12468365 10.1054/clnu.2002.0585

[30] Hebuterne X, Lemarie E, Michallet M, de Montreuil CB, Schneider SM, et al (2014) Prevalence of malnutrition and current use of nutrition support in patients with cancer. JPEN J Parenter Enteral Nutr 38: 196–204. doi: 10.1177/0148607113502674 24748626 10.1177/0148607113502674

[31] Daniele A, Divella R, Abbate I, Casamassima A, Garrisi VM, et al (2017) Assessment of Nutritional and Inflammatory Status to Determine the Prevalence of Malnutrition in Patients Undergoing Surgery for Colorectal Carcinoma. Anticancer Res 37: 1281–1287. doi: 10.21873/anticanres.11445 28314293 10.21873/anticanres.11445

[32] Li F, Liu YW, Wang XF, Liu GW (2014) Evaluation of malnutrition in patients with nervous system disease. Expert Rev Neurother 14: 1229–1237. doi: 10.1586/14737175.2014.957184 25192880 10.1586/14737175.2014.957184

