# Supplementary material for: A comprehensive nutritional survey of hospitalized patients: Results from nutritionDay 2016 in China
Source: PLoS One. 2018 Mar 22;13(3):e0194312. doi: 10.1371/journal.pone.0194312 (PMC5863998; doi:10.1371/journal.pone.0194312)
Supplement: S1 Table — (PDF) [file pone.0194312.s001.pdf]

Please mark the correct boxes that apply to this patient

nutritionDay worldwide

ABOUT YOUR PATIENT
SHEET 2a

Date ☐☐ ☐☐ ☐☐☐☐
Center-Code ☐☐☐☐☐
Unit-Code ☐☐☐☐☐

Patient number ☐☐☐ **Patient Initials** ☐☐☐☐ **Year of birth** ☐☐☐☐

Sex ○ Female ○ Male **Date of admission** ☐☐ ☐☐ ☐☐☐☐

Weight ☐☐☐ lb. ○ estimated ○ measured

Height ☐ ft. ☐ in. ○ estimated ○ measured **Patient consent** ○ Yes ○ No

PLEASE CONTINUE ONLY IF PATIENT GAVE CONSENT!

1. This hospital admission was… ○ **planned** ○ **an emergency** ○ **I do not know**

2a. Diagnosis at admission (mark all that apply)

- ☐ 0100 Infectious and parasitic diseases
- ☐ 0200 Neoplasms
- ☐ 0300 Blood and bloodforming organs and the immune mechanism
- ☐ 0400 Endocrine, nutritional and metabolic diseases
- ☐ 0500 Mental health
- ☐ 0600 Nervous system
- ☐ 0700 Eye and adnexa
- ☐ 0800 Ear and mastoid process
- ☐ 0900 Circulatory system
- ☐ 1000 Respiratory system
- ☐ 1100 Digestive system
- ☐ 1200 Skin and subcutaneous tissue
- ☐ 1300 Musculoskeletal system and connective tissue
- ☐ 1400 Genitourinary system
- ☐ 1500 Pregnancy, childbirth and the puerperium
- ☐ 1600 Conditions originating in the perinatal period
- ☐ 1700 Congenital/chromosomal abnormalities
- ☐ 1800 Symptoms, signs, abnormal clinical/lab findings
- ☐ 1900 Injury, poisoning
- ☐ 2000 External causes of morbidity and mortality (e.g. transport accidents, assaults)
- ☐ 2100 Factors influencing health status and contact with health services

2b. Main reason for admission (choose only one code from above) ☐☐☐☐

3. Which conditions/comorbidities does this patient have? (mark an answer for each)

Condition			Condition		
Cardiac insufficiency	○ Yes	○ No	Diabetes	○ Yes	○ No
Myocardial infarction	○ Yes	○ No	Cancer	○ Yes	○ No
Chronic lung disease	○ Yes	○ No	Infection	○ Yes	○ No
Cerebral vascular disease	○ Yes	○ No	Dementia	○ Yes	○ No
Peripheral vascular disease	○ Yes	○ No	Major depressive disorder	○ Yes	○ No
Chronic liver disease	○ Yes	○ No	Other chronic mental disorder	○ Yes	○ No
Chronic kidney disease	○ Yes	○ No	Other chronic disease	○ Yes	○ No

4a. Previous operation during this hospital stay
○ Yes, planned ○ No
○ Yes, acute
days since operation ☐ days

4b. Planned operation during this hospital stay
○ Yes, today or tomorrow
○ Yes, later
○ No

5. Previous ICU admission during this hospital stay? ○ Yes ○ No

6. Is this patient terminally ill? ○ Yes ○ No ○ I do not know

7. Fluid status (TODAY) ○ Normal ○ Overloaded ○ Dehydrated ○ I do not know

8. Number of different medications planned (TODAY) **oral** ☐☐ **other** ☐☐

9. Was this patient identified as malnourished or at risk of malnutrition?
○ Malnourished ○ At risk ○ No ○ I do not know

Please continue with Sheet 2b

© nutritionDay - Authors: Hiesmayr / Schindler / Simon / Kiss / Kosak / Bauer - Medical University of Vienna / ESPEN Version 1 - 07.10.2016

Please mark the correct boxes that apply to the patient

nutritionDay worldwide

ABOUT YOUR PATIENT SHEET 2b

Date ☐☐☐☐☐☐☐☐

Center-Code ☐☐☐☐☐

Unit-Code ☐☐☐☐☐

Patient number ☐☐☐ **Patients Initials** ☐☐☐☐

10. IV Fluids (TODAY) ☐ Electrolyte solution (NaCl, Ringers lactate, etc) ☐ 5% Glucose solution

11. Number of ONS drinks planned (TODAY) ☐☐

12. Nutrition intake (TODAY) (mark an answer for each)

Regular hospital food	◯ Yes	◯ No	◯ I do not know
Fortified/enriched hospital food	◯ Yes	◯ No	◯ I do not know
Protein/energy supplement (e.g. ONS drinks)	◯ Yes	◯ No	◯ I do not know
Enteral nutrition	◯ Yes	◯ No	◯ I do not know
Parenteral nutrition	◯ Yes	◯ No	◯ I do not know
Special diet	◯ Yes	◯ No	◯ I do not know

13a. All lines and tubes (TODAY) (mark an answer for each)

Central Venous	◯ Yes	◯ No	Nasoduodenal	◯ Yes	◯ No
Peripheral venous access	◯ Yes	◯ No	Enterostoma	◯ Yes	◯ No
Nasogastric	◯ Yes	◯ No	Percutaneous endoscopy/surgical gastrostomy	◯ Yes	◯ No
Nasojejunal	◯ Yes	◯ No	Percutaneous endoscopy/surgical jejunostomy	◯ Yes	◯ No

13b. Were there complications with nutrition related lines and tubes since admission? (infections /obstructions)

◯ Yes, previously ◯ Yes, ongoing ◯ No ◯ I do not know

14. Please indicate if any of the following was done for this patient since admission (mark an answer for each)

Energy requirements were determined	◯ Yes	◯ No	◯ I do not know
Protein requirements were determined	◯ Yes	◯ No	◯ I do not know
Food/Nutrition intake was recorded in the patient record	◯ Yes	◯ No	◯ I do not know
Nutrition treatment plan was developed	◯ Yes	◯ No	◯ I do not know
Nutrition expert was consulted	◯ Yes	◯ No	◯ I do not know
Malnutrition status is recorded in the patient record	◯ Yes	◯ No	◯ I do not know

15a. Energy goal (YESTERDAY)

- ◯ <500 kcal
- ◯ 500-999 kcal
- ◯ 1000-1499 kcal
- ◯ 1500-1999 kcal
- ◯ >=2000 kcal
- ◯ Not determined
- ◯ I do not know

OR please insert ☐ kcal/lb

15b. Energy intake (YESTERDAY)

- ◯ <500 kcal
- ◯ 500-999 kcal
- ◯ 1000-1499 kcal
- ◯ 1500-1999 kcal
- ◯ >=2000 kcal
- ◯ Not determined
- ◯ I do not know

OR please insert ☐ kcal/lb

16. Since admission, this patient's health status has…

- ◯ Improved
- ◯ Deteriorated
- ◯ Remained the same
- ◯ This patient has just been admitted
- ◯ I do not know

Thank you!
