# Supplementary material for: A comprehensive nutritional survey of hospitalized patients: Results from nutritionDay 2016 in China
Source: PLoS One. 2018 Mar 22;13(3):e0194312. doi: 10.1371/journal.pone.0194312 (PMC5863998; doi:10.1371/journal.pone.0194312)
Supplement: S2 Table — (PDF) [file pone.0194312.s002.pdf]

Please mark the correct boxes

nutritionDay worldwide

PATIENT SHEET
SHEET 3a

Date
Center-Code
Unit-Code

Patient number | Patients Initials

1. What are your typical dietary habits? (mark all that apply)

- ☐ No special dietary habits
- ☐ I am vegetarian
- ☐ I adhee to a vegan diet
- ☐ I eat gluten-free diet
- ☐ I avoid added sugars
- ☐ I avoid carbohydrates
- ☐ I eat a low fat-diet
- ☐ I am lactose intolerant
- ☐ Other special diet due to intolerances/allergies
- ☐ Other

2. Where did you live before your current hospital admission?

- ◯ At home
- ◯ In a nursing home or other live-in facility
- ◯ I was transferred from another hospital
- ◯ Other

3. In general, are you able to walk?

- ◯ Yes
- ◯ Yes, with someone's help
- ◯ Yes, independently using a cane, walker, or crutches
- ◯ No, I have a wheelchair
- ◯ No, I am bedridden

4. In general, how would you say your health is?

◯ Very good ◯ Good ◯ Fair ◯ Poor ◯ Very poor

5. Over the last 12 months prior to your current hospital admission approximately…

… how many times have you seen a doctor? ☐ times

… how many times have you been admitted to the hospital (Emergency room, any ward)? ☐ times

… how many nights in total have you spent in hospital? ☐ nights

6. How many different medications do you take routinely each day (prior to hospitalisation)?

- ◯ 1-2
- ◯ 3-5
- ◯ More than 5
- ◯ None
- ◯ I do not know

7. Do you have health insurance?

- ◯ Yes, private insurance only
- ◯ Yes, public insurance only
- ◯ Yes, both
- ◯ No
- ◯ I prefer not to answer

8. What was your weight 5 years ago? ☐ lb. ◯ I do not know

9a. Have you lost weight within the last 3 months?

- ◯ Yes, intentionally
- ◯ Yes, unintentionally
- ◯ No, my weight stayed the same
- ◯ No, I gained weight
- ◯ I do not know

9b. If yes, how many kg did you lose? ☐ lb. ◯ I do not know

10. Did you know about your hospitalisation two days before admission? ◯ Yes ◯ No

11. Please indicate if you …

... were weighed at admission	◯ Yes	◯ No	◯ I do not know
... were informed about your nutrition status	◯ Yes	◯ No	◯ I do not know
... were informed about nutrition care options	◯ Yes	◯ No	◯ I do not know
… received special nutrition care	◯ Yes	◯ No	◯ I do not know

Please continue with Sheet 3b

Please mark the correct boxes that apply to you

nutritionDay worldwide

PATIENT SHEET
SHEET 3b

Date ☐☐☐☐☐☐☐☐

Center-Code ☐☐☐☐

Unit-Code ☐☐☐☐

Patient number ☐☐☐ **Patients Initials** ☐☐☐☐

12. How well have you eaten in the week before you were admitted to the hospital?
- ○ More than normal
- ○ Normal
- ○ About 3/4 of normal
- ○ About half of normal
- ○ About a quarter to nearly nothing

13. In general, how satisfied are you with the food at the hospital?
- ○ Very satisfied
- ○ Somewhat satisfied
- ○ Neutral
- ○ Dissatisfied
- ○ Very dissatisfied
- ○ I do not know

14. Did you get any help with eating TODAY?
- ○ Yes, from family or friends
- ○ Yes, from hospital staff
- ○ No

15. Were you able to eat without interruption TODAY?
- ○ Yes
- ○ No

16a. Please indicate how much hospital food you ate for lunch or dinner TODAY:

about all 1/2 1/4 nothing

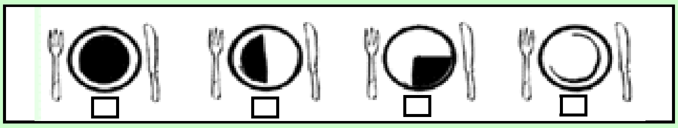

16b. The portion size of the meal I ordered TODAY was…
- ○ standard
- ○ smaller
- ○ larger
- ○ I do not know

17. If you did not eat everything of your meal, please tell us why: (mark all that apply)
- ☐ I did not like the type of food offered
- ☐ I did not like the smell/taste of the food
- ☐ The food did not fit my cultural/religious preferences
- ☐ The food was too hot
- ☐ The food was too cold
- ☐ Due to food allergy/intolerance
- ☐ I was not hungry at that time
- ☐ I do not have my usual appetite
- ☐ I have problems chewing/swallowing
- ☐ I normally eat less than what was served
- ☐ I had nausea/vomiting
- ☐ I was too tired
- ☐ I cannot eat without help
- ☐ I was not allowed to eat
- ☐ I had an exam, surgery, or test and missed my meal
- ☐ I did not get requested food

18. Enter the number of glasses/cups of the drinks you consumed in the last 24 hours

☐ Water	☐ Coffee	☐ Fruit juice	☐ Nutrition drink
☐ Tea	☐ Milk	☐ Soft drinks	☐ Other

19a. Did you eat any food apart from hospital food TODAY? ○ Yes ○ No

19b. If yes, what did you eat?
- ☐ Sweet snacks
- ☐ Salty snacks
- ☐ Homemade food
- ☐ Fruits
- ☐ Dairy products
- ☐ Food delivered/restaurant
- ☐ Sandwich
- ☐ Other

20. How has your food intake changed since your hospital admission?

○ Increased ○ Decreased ○ Stayed the same ○ I do not know

21. TODAY I feel…
- ○ Stronger than at admission
- ○ Weaker than at admission
- ○ Same as at admission
- ○ I was admitted today
- ○ I do not know

22. Can you walk without assistance TODAY?
- ○ Yes
- ○ No, only with assistance
- ○ No, I stay in bed

23. Did anyone help you complete this questionnaire? ○ Yes ○ No

THANK YOU!
