# Supplementary material for: A comprehensive nutritional survey of hospitalized patients: Results from nutritionDay 2016 in China
Source: PLoS One. 2018 Mar 22;13(3):e0194312. doi: 10.1371/journal.pone.0194312 (PMC5863998; doi:10.1371/journal.pone.0194312)
Supplement: S3 Table — (PDF) [file pone.0194312.s003.pdf]

Patients list and outcomes (all ND-patients)

nutritionDay worldwide

30 days OUTCOMES

Date

Center-Code

Unit-Code

Patient Number	Patient Initials	Discharge Date	Discharge Diagnosis	Outcome	Readmitted since ND
…or patient sticker			(see box 1. for codes)	(see box 2. for codes)	(see box 3. for codes)

1. Either use full ICD-10 codes or the codes below

Enter up to 6 codes, in the same order as in your records

0100 Infectious and parasitic diseases
0200 Neoplasms
0300 Blood and bloodforming organs and the immune mechanism
0400 Endocrine, nutritional and metabolic diseases
0500 Mental health
0600 Nervous system
0700 Eye and adnexa
0800 Ear and mastoid process
0900 Circulatory system
1000 Respiratory system
1100 Digestive system
1200 Skin and subcutaneous tissue
1300 Musculoskeletal system and connective tissue
1400 Genitourinary system
1500 Pregnancy, childbirth and the puerperium
1600 Conditions originating in the perinatal period
1700 Congenital/chromosomal abnormalities
1800 Symptoms, signs, abnormal clinical/lab findings
1900 Injury, poisoning
2000 External causes of morbidity and mortality (e.g. transport accidents, assaults)
2100 Factors influencing health status and contact with health services

2. Outcome Code

1= Still in the hospital
2= Transferred to another hospital
3= Transferred to long term care
4= Rehabilitation
5= Discharged home
6= Death
7= Others

3. Readmission Code

1= No
2= Yes, same hospital planned
3= Yes, same hospital unplanned
4= Yes, different hospital planned
5= Yes, different hospital unplanned
6= Unknown

THANK YOU!
